# Male partner influence on family planning and contraceptive use: perspectives from community members and healthcare providers in KwaZulu-Natal, South Africa

**DOI:** 10.1186/s12978-019-0749-y

**Published:** 2019-06-25

**Authors:** Yolandie Kriel, Cecilia Milford, Joanna Cordero, Fatima Suleman, Mags Beksinska, Petrus Steyn, Jennifer Ann Smit

**Affiliations:** 1 MatCH Research Unit (Maternal, Adolescent and Child Health Research Unit), Department of Obstetrics and Gynaecology, Faculty of Health Science, University of the Witwatersrand, Durban, South Africa; 2 School of Public Health and Nursing, College of Health Science, University of KwaZulu-Natal, Durban, South Africa; 3 Discipline of Pharmaceutical Science, College of Health Science, University of KwaZulu-Natal, Durban, South Africa; 4 Department of Reproductive Health and Research, World Health Organisation (WHO), Geneva, Switzerland

**Keywords:** Contraception, Family planning, South Africa, Male partners, Influencers, Barriers, Facilitators

## Abstract

**Background:**

South Africa faces numerous reproductive challenges that include high rates of unplanned and adolescent pregnancies. The uptake and utilization of family planning services and modern contraception methods depend on numerous factors. The male partner plays a key role in reproductive health but data on this topic are outdated or have a predominant HIV prevention focus. The purpose of this paper is to explore the influence of male partners on family planning and contraceptive (FP/C) uptake and use within the contemporary South African setting, and to identify further areas of exploration.

**Methods:**

This qualitative study was conducted in a community and healthcare provision setting in the eThekwini District in KwaZulu-Natal province, South Africa. Data were collected from twelve community-based focus group discussions (*n* = 103), two healthcare providers focus group discussions (*n* = 16), and eight key informant individual in-depth interviews. Following a constructionist paradigm and using the health utilization behaviour model, data were analysed using thematic analysis, allowing a robust and holistic exploration of the data.

**Results:**

The data from this study revealed the complex and evolving role that male partners play in FP/C uptake and use within this setting. Key themes from the data elucidated the dual nature of male involvement in FP/C use. Culturally influenced gender dynamics and adequate understanding of FP/C information were highlighted as key factors that influenced male attitudes and perceptions about contraceptive use, whether positively or negatively. Male opposition was attributed to limited understanding; misunderstandings about side-effects; male dominance in relationships; and physical abuse. These factors contributed to covert or discontinued use by female partners. Pathways identified through which male partners positively influenced FP/C uptake and access include: social support, adequate information, and shared responsibility.

**Conclusions:**

Understanding the role that male partners play in FP/C uptake and use is important in preventing unintended pregnancies and improving family planning policy and service delivery programmes. By identifying the barriers that male partners present, appropriate strategies can be implemented. Equally important is identifying how male partners facilitate and promote adherence and use, and how these positive strategies can be incorporated into policy to improve the uptake and use of FP/C.

## Plain English summary

South Africa faces numerous reproductive health challenges including high unmet need among unmarried women, high rates of unplanned pregnancies, and high rates of adolescent pregnancies. Male partners play a key role in reproductive health, but more information is needed to gain a better understanding about the influence of male partners on the uptake of family planning and contraceptive (FP/C) use within the contemporary South African setting. Numerous pathways were identified that showed how the male partner influences FP/C uptake and use. Male partners can influence FP/C use negatively by obstructing contraceptive use that result in either discontinuation or covert use. Both discontinued and covert use increases the female partner’s risk of having an unplanned or unintended pregnancy. The data also showed that male partners can improve FP/C uptake and use by providing social support, supplying FP/C information and sharing the responsibility of using FP/C correctly and consistently.

Key factors that require attention are the quality of FP/C information given to men, the inclusion of men in FP/C programmes, and the effect of the decline in marriage rates in this setting on FP/C uptake and use. Using a qualitative and community-based approach contextualised the findings and identified further areas for research.

## Background

Unmet need for contraception remains a global challenge and in 2014, it wa estimated that more than 225 million women in the developing world were unable to access and use family planning or contraception (FP/C) [1]. While globally there has been an increase in contraceptive prevalence and decrease in unmet need since 1970, the Sub-Saharan Africa region continues to have the lowest contraceptive prevalence at 24% and highest level of unmet need at 25% [2].

South Africa faces key reproductive health challenges that are entrenched in socio-political and cultural factors. The overall unmet need is 18%, while the contraceptive prevalence rate (CPR) for married women is 54 and 64% for unmarried women [3]. Currently there is no data available on male unmet need in South Africa, however the couple year protection rate (CYPR) is estimated to be 70.2% [4]. SA’s CPR and unmet need rates have stagnated over the past two decades [5, 6]. This stagnation is a cause for concern as modern FP/C methods and services are freely and widely available through the SA public healthcare system [7].

Along with the stagnated CPR and high unmet need, other key reproductive health challenges include high rates of unplanned pregnancies, high rates of adolescent pregnancies, and a large generalised HIV epidemic where young women are predominantly affected [3, 8, 9]. Unplanned pregnancies tend to be highest among young, unmarried women who are HIV positive [9, 10]. Child bearing also starts at a very young age for women in South Africa, with the average age of first conception being below 21 years of age, with those women being unmarried [11]. Unplanned pregnancies amongst this young age group, between 15 and 25 years, are particularly worrisome as studies have demonstrated that there is a potential link between HIV acquisition, antiretroviral treatment initiation and unplanned pregnancies amongst young women [9, 12, 13].

Numerous factors influence FP/C use including inter-personal relationships [2]. Inter-personal relationships include family, community members, religious leaders, healthcare providers and intimate partners [6, 14]. The most significant inter-personal relationship in FP/C use is the intimate male partner relationship and the role of gender dynamics [15, 16]. The male partner’s role in FP/C use is complex, and ranges from macro-level socio-cultural, economic, political and gendered factors, to more micro everyday-level factors [2, 14, 17].

Even though nearly 40% of households in South Africa are headed by women, men continue to hold considerable power over women [7, 11]. This is mainly attributed to the culturally elevated status that men have over women and men being more economically empowered [11, 14]. Male dominance is reinforced through political and economic mechanisms that limit women’s access to financial independence and therefore their ability to access and use FP/C methods and services [2, 14, 17–19]. In the South African context, gender inequality, past political policies, and patriarchal cultural norms drive female disempowerment, which negatively influences FP/C use [7, 16, 20].

Culture guides behaviour and is a macro-structural factor that influences reproductive behaviour [21–23]. Patriarchal views on gendered roles are socially constructed and reinforced within various cultural settings and result in women lacking autonomy to make their own decisions about using FP/C methods [24]. These patriarchal cultural views give men power to decide how many children a couple should have [25].

Recent studies in the sub-Saharan region have identified a variety of micro-level factors through which the male partner negatively influences FP/C uptake and use. These factors include male partners having negative personal beliefs about FP/C; limited access to FP/C information; myths and misconceptions; perceived side effects including decreased sexual pleasure; marital status; poor economic status; religious influences; limited male contraceptive choice; suspicion of female partner infidelity; and male preference for larger families as reasons to oppose FP/C use [6, 14, 16, 20, 26–28]. Furthermore, negative interactions with healthcare providers (HCPs) is another important factor that influence male involvement in FP/C use [29].

Most FP/C studies are conducted from the female perspective and focus on women who are clinic attendees, to capture the FP/C experience. This reflects the female dominated view that is often captured in FP/C studies and results in the male voice being silenced [16, 28]. There is also an overbearing assumption that men are always barriers, uninterested or by-standers in FP/C use, [30, 31] and that their influence results in discontinuation of FP/C use.

Studies have shown that good communication between couples positively influence FP/C use, and can reduce the risk of misconceptions [6]. Good communication also results in joint-decision making about FP/C use, which has been linked to improved adherence [27]. Supportive male partner attitudes and positive views of FP/C services play an important role in promoting FP/C use [15]. It has also been reported that men exposed to FP/C educational programmes were four times more likely to support FP/C use [32].

Much of the South African work focussing primarily on FP/C is outdated, due to the shift in focus to HIV/AIDS treatment and prevention, and the integration of care [33]. Furthermore, most recent studies are conducted outside the South African setting, focussing on areas where unmet need for FP/C is comparatively high. While these findings are enlightening, they are in some instances more relevant within their own context. It is therefore important to examine context specific behaviours when studying a complex topic such as FP/C use, as these practices are steeped in local cultural understandings, beliefs and norms [14].

The influence that male partners has on FP/C use varies across regions and settings. Clarity is needed to understand the holistic role of male partners in FP/C use within specific contexts, especially the contemporary South African setting [17, 31, 34]. In this article, we explore the role that male partners play in FP/C uptake and use from a community and healthcare provider-based perspective.

### Context

The eThekwini District, where the data for this study was collected, is the third largest in South Africa, with a population size of slightly below three and a half million people, the dominant language spoken is IsiZulu [35]. The CYPR for the district is 66.1% for 2016/2017, which is just above the national target of 50% [4]. The province has seen the largest decrease in CYPR of 8 percentage points between 2014/2015 and 2016/2017 [4]. KwaZulu-Natal has the third lowest CPR for any method at 53.1% in South Africa.

In terms of demographics, currently, 23.9% are married, 66% have never married, 5.6% are living together, 2.9% are widowed, 1% is divorced and 0.5% separated [35]. The low marriage rates in the district reflect the current state of marriage in South Africa that is in decline and at a national low [36, 37]. KwaZulu-Natal also has the highest number of people living with HIV/AIDS in the country [38].

Two large areas were chosen within the district that represent a mixture of rural, peri-urban and urban areas. Area 1 (defined as the rural area for purposes of this study) presents a combination of rural/peri-urban economic mixture with approximately 60.8% of the houses being formal, while Area 2 represents the urban/peri-urban setting with 65.1% of houses being formal. Both areas are predominantly populated by Black South Africans (99.5%), who speak IsiZulu [35].

## Methods

This study was conducted as part of formative work to inform the development of an intervention that aimed to increase met need for FP/C through community and healthcare provider (HCP) participation, in South Africa, Kenya and Zambia (the UPTAKE Project). In this paper, we report on findings from the South African site on the role that men play in FP/C uptake and use.

A qualitative methodology was used to gather data from the two areas described above within the eThekwini district of KwaZulu-Natal. In-depth interviews (IDIs) were conducted with key informants (KIs) and focus group discussions (FGDs) were held with community members and HCPs between June and December 2015. A key aspect of this study was the inclusion of community members, who may or may not have been users of FP/C, rather than clients accessing services at healthcare facilities. The rationale for this approach was to obtain a contemporary perspective on FP/C services and method use by community members. This information could then inform FP/C services to improve FP/C delivery to those who are at need.

In-depth interviews (IDIs) were conducted with key informants (KIs), who ranged from educators to community care givers, traditional healers, and programme managers for sexual and reproductive health programs. Eight KIs were selected purposively or via snowball sampling, based on expertise, and participated in an IDI. It was estimated that between eight and ten KI IDIs would be required to reach data saturation, which was achieved with the eight KIs interviewed.

Healthcare providers from eight healthcare facilities in the district were invited to participate. Two FGDs were held with HCPs who were directly providing FP/C services or who were based in service delivery points which may promote or inform women about FP/C options. Healthcare providers from eight healthcare facilities in the district were invited to participate. In total sixteen HCPs participated in the FGDs. HCP group 1 (*n* = 8) consisted of higher ranked professionals, such as professional nurses and operational managers. HCP group 2 (n = 8) consisted of counsellors, nursing assistants and enrolled nurses. The HCP groups were structured in such a way as to promote open discussion.

Finally, twelve FGDs were conducted with male and female community members (*N* = 103). The groups were composed according to several factors: urban/peri-urban vs rural/peri-urban, age, sex, parity and marital/relationship status. Local community advisory boards (CAB) assisted with identifying potentially eligible participants and purposive sampling was used to recruit community members according to FGD categories and eligibility criteria. (See Table 1 for participant breakdown.)

**Table 1:** Breakdown of participants per FGD and IDI participants

FGDs conducted	No. of participants (n)
1. Females, urban, teenagers (aged 15–19 years)	9
2. Females, rural/peri-urban, teenagers (aged 15–19 years)	10
3. Females, urban, young adults (aged 20–34 years)	8
4. Females, rural/peri-urban, young adults (aged 20–34 years)	10
5. Females, urban, adults (aged 35–49 years)	8
6. Females, rural/peri-urban, adults (aged 35–49 years)	7
7. Males, teenagers (aged 15–19 years)	10
8. Males, young adults (aged 20–34 years)	8
9. Males, adults (aged 35–49 years)	7
10. Females who are unmarried, single (20–34 years)	8
11. Females who are married/in a relationship > 1-year (20–34 years)	10
12. Females with no children (who are not infertile) (18–49 years)	8
Total community participants	103
13. HCP from local health facilities (including management, professional nurses): Group 1	8
14. HCP from local health facilities (including enrolled nurses, counsellors, and other operational staff): Group 2	8
Total HCP participants	16
Key stakeholders	
1. Education	1
2. Community Care Givers	2
3. Traditional Healer	1
4. Programme Managers working in sexual and reproductive health (SRH)	4
Total key stakeholders	**8**

The FGDs were conducted by research assistants who were matched by gender and language to the participants to facilitate rapport. The research assistants were all trained in conducting FGDs and all had previous experience of using qualitative methodology to collect data. HCP FGDs were conducted by the study project manager who is fluent in both English and isiZulu. The FGDs were conducted in either isiZulu and/or English to ensure that participants were able to express their views without a language barrier. FGDs lasted between one to two hours.

The IDIs were conducted by either a research assistant, study coordinator, or senior researcher to facilitate rapport with the participants. FGDs were conducted at community-based facilities. Key stakeholders were interviewed at locations convenient for them, that ranged from the research site’s offices to the participant’s home.

All the FGDs and IDIs that were conducted in isiZulu were translated and back-translated by research assistants who are fluent in both languages. These transcripts were then reviewed and checked for accuracy by the researchers. Any ambiguity in the translations were discussed and clarified with the respective research assistant and interviewer to ensure the accuracy of the translations. At the end of the project, the results were shared with the community members, and there was a high degree of agreement from the community members with the results presented. This further contributed to the validity and accuracy of the data.

Interview guides contained key theme specific questions that were tailored for each category type of participant, including: the female FGDs, male FGDs, HCP FGDs, and key stakeholder IDIs. Adolescent participants were asked the same questions as per their respective gender to the adult participants. Similar key theme questions were asked that inquired about understandings of family planning; knowledge, attitudes and practices; key barriers and enablers to family planning access; perceptions and definitions of quality of care; and the role of community participation in family planning and contraceptive services. Specifically, participants were asked who the most important people are in supporting women and girls in choosing and using FP/C. This resulted in the emergence of the male partner and themes described in this paper.

The purpose of the approach discussed above was to gain the perspectives of each category/type of participant. Obtaining various perspectives is congruent with employing a social constructionist approach in analysing the data, where opinions are valued equally. This was an important stance to adopt considering the varied categories of participants that were involved in this study.

Voluntary informed consent was obtained from all adult participants aged 18 years and older. For minors, below the age of 18 years, consent was obtained from their parents and/or legal guardians and assent from the minors to participate in the study. Permission to audio record all FGDs and IDIs was included in the informed consent forms. In addition to the audio recordings, detailed field notes were taken. During FGDs the interviewer was assisted by a note-taker who was fluent in the language the FGD was conducted in. IDI interviewers took their own notes while conducting the interview.

### Data analysis

The IDIs and FGDs were audio recorded and transcribed verbatim. The transcripts of the FGDs and IDIs conducted in isiZulu were translated into English. Demographic data were collected and descriptively analysed.

Qualitative data analysis was done using thematic content analysis, following a social constructionist approach. Social constructionism provided a theoretical framework through which to explain and understand the interpretation of cultural constructs in family planning and contraceptive use [39, 40]. These cultural constructs provide mechanisms of control that govern individual’s behaviour within a given society, which is pertinent to the study of sexual and reproductive health [23]. In addition, Andersen’s [41–43] Health Utilization Model and Penchansky and Thomas’s [44] definition of the concept of access to healthcare further aided in the development of the a priori code list.

A master code book was developed amongst the researchers in all three countries in which the larger study was conducted. This team approach allowed for rich discussion about the meaning of concepts and codes, further establishing the validity and appropriateness of the code list. Independent coders double coded a subset of the data to increase reliability of the data. The constant comparison method was used to further explore the data and develop additional themes [45]. NVivo (version 10, QSR International) was used as the computer assisted qualitative data analysis software that facilitate coding and analysis of the data.

A priori themes were identified using the Health Utilization Model, the Bruce-Jain framework for Quality of Care, and Penchansky and Thomas’s definition of access to healthcare [41, 44, 46]. Inductive and emergent themes were also elucidated in the data of which the male partner’s role in FP/C uptake and use emerged. The use of social constructionism as a theoretical framework allowed for the exploration of opinions about FP/C use by a variety of different perspectives. This stance allowed for a more robust and richer understanding to emerge about the role that male partner’s play in FP/C use in this setting.

Themes were derived from the initial coding, and later grouped into categories. The constant comparison method as described by Ryan and Bernard (2003) [45] was used to identify emergent themes in the data. An in-depth discussion on the overall methodology used in this project is described elsewhere [47].

## Results

### Demographic results

Table 2 shows the demographic details of the community members, and Table 3 shows HCP and key informant demographics. Community males had a mean age of 23.8 years, compared to females who had a mean age of 26.4 years. Notably, only two female participants were married. Of the male and female participants who reported pregnancies, 32 and 66.6% respectively, 87.5% of males and 86.5% of females reported that their pregnancies were unplanned. For current contraceptive use, 12% of males and 19.2% of females reported not using any methods at the time of data collection.

**Table 2:** Demographic data for male and female community participants

Demographic Characteristics	Community males (*n* = 25)		Community females (*n* = 78)	
	Percent %	N	Percent %	N
Age	23.8 years [15–40]		26.4 years [15–49]	
Female	–		75.0 (n = 78)	103
Male	24.27	103	–	
Education				
Below completed secondary school level	84.0	21	48.7	38
Above completed secondary school level	16.0	4	51.3	40
Relationship status				
Regular partner, > 1 yr., not living together	28.0	7	62.0	48
Regular partner, < 1 yr., not living together	28.0	7	21.0	16
Regular partner, > 1 yr., living together	8.0	2	3.0	2
Married	0	0	3.0	2
Divorced	0	0	1.0	1
Casual partner	16.0	4	0	0
No current partner	4.0	1	10.0	8
Multiple partners	16.0	4	0	0
Sexual and reproductive history				
Positive Pregnancy	32.0	8	66.6	52
Unplanned pregnancies	87.5	7	86.5	45
Planned pregnancies	12.5	1	15.3	8
Current contraceptive use				
None	12.0	3	19.2	15
Male condoms	68.0	17	60.2	47
3 monthly injection	24.0	6	24.0	19
Pill	8.0	2	2.5	2
Implant	4.0	1	8.9	8

**Table 3:** Demographic data for the healthcare providers and key informants

Demographic	HCP group 1 (*n* = 8)		HCP group 2 (*n* = 8)		Key informants (*n* = 8)	
	Percent %	N		N	Percent %	N
Age	39.2 years [28–56]		37.6 years [26–47]		51.2 years [25–66]	
Female	75.0	6	100.0	8	87.5	7
Male	25.0	2	0	0	12.5	1
Education						
Below completed secondary school level	0	0	0	0	25.0	2
Above completed secondary school level	0	0	0	0	12.5	1
Tertiary incomplete	0	0	37.5	3	0	0
Tertiary complete	100	8	62.5	5	62.5	5
Years of experience in current position	4.9 years		6.4 years		10 years	

### Thematic results

**Fig. 1:**
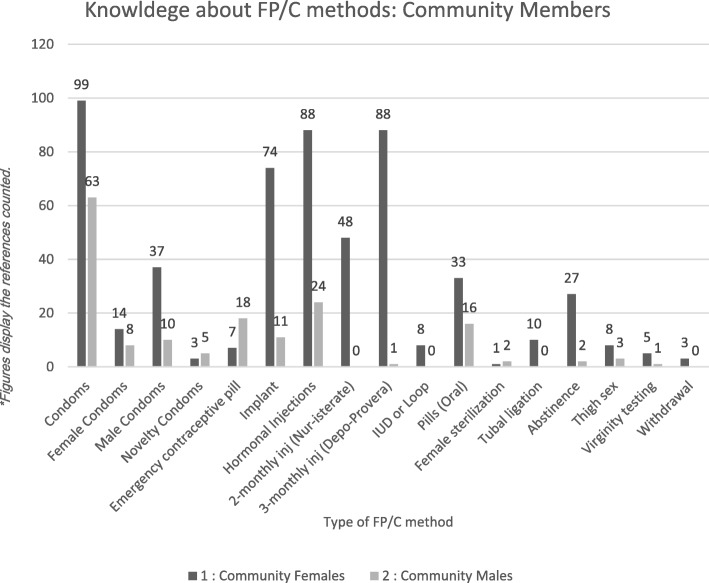
Knowledge about FP/C Methods: Community Members

Figure 1 below demonstrates the various FP/C methods that community participants discussed. Community members had a good range of information about available products. Figure 1 also demonstrates the differences and similarities in FP/C method discussion between men and women. Male condoms were equally discussed between men and women, followed by discussions about hormonal injections. Perhaps the most interesting aspect to note of Fig. 1 is that men discussed emergency contraception significantly more than women. This issue will be addressed further in a separate paper.

### The obstructive male partner

Knowledge about the various FP/C methods available in the community was high among community members (Fig. 1). Even so, some young male adult participants reported that they required more detailed information to improve their understanding of FP/C methods:

*F: “[O] kay, what information does he [the male partner] need about these methods?*

*P1: “It’s important to know how it works. Concerning my health, if there will be any effect and how is going to help me?”*

*P4: “How safe is it [for] me, and my partner [?] … It is right for my partner while it gives me a problem. If my partner has been using this thing, [ … ] I shouldn’t have a problem afterwards.”*

*P8: “Yes, I want to know a lot about them because I don't know much about it.”*

*[Young Adult Males, FGD]*

HCPs pointed out that the feminisation of FP/C services and education contributed to men being misinformed:

*“Because now we are not concentrating on males we only give them condoms. So it ends up being like, feminine, yet it’s not supposed to be, it’s a family thing it’s [for] everybody.”*

*[Female KI, HCP, IDI]*

Some female participants argued that a lack of information and understanding could result in incorrect assumptions about side-effects and negative attitudes towards FP/C by male partners, as well as misconceptions and myths about the effects of using FP/C methods:

*“Males are not the same, there are males who will hear females talking among themselves saying someone who takes injection becomes wet, he takes that [information, and when] you tell him that you [use] the injection he says you [are] wet [ … ] what does he know, what does he know?”*

*[Young female adult, Rural, FGD]*

A married female participant added to the view the men do not understand how FP/C methods work:

*“He believes that if you are on injection you are killing the baby because he does not have babies [ … ] It means that you go to the clinic to kill [the] sperm that he produces.”*

*[Married Females, FGD]*

Side effects, whether perceived or real, were identified as key contributors to male opposition to FP/C use. Although numerous side effects are associated with FP/C use, increased vaginal wetness and decreased male sexual pleasure were the two most significant side effects described by men.

One young male adult participant explained how the use of the hormonal injection and the associated increased vaginal wetness was unacceptable for men:

*“Ay and you find that the queen [female partner] ... you find that your queen is loose like jelly [all laughing] and that is caused by the injection. That is not right my brother that thing [all laughing and one says: it does not treat her right]. The injection is wrong.”*

*[Young Adult Males, FGD]*

The view that men over exaggerated vaginal wetness was shared widely across the female participant groups, as one female participant explains:

*“I agree with [other participant], most males can see that you are on injection. There is nothing that they think about besides water retention that you are always wet. Even if you are not he will say you are wet.”*

*[Married Females, FGD]*

Decreased male sexual pleasure was another side effect attributed to male opposition to FP/C use:

*“[S] ometimes we men don’t really allow women to use … these family planning services [...] Because sometimes they make us uncomfortable and they make sex, less enjoyable.”*

*[Male KI, Edu, IDI]*

This was particularly in reference to male condoms, as one young adult male participant pointed out:

*“[U] nprotected sex is nice [others laughing] and this is something that makes men happy. Ay, unprotected sex is nice. With a condom, you just feel the plastic [..]”*

*[Young Adult Males, FGD]*

Men could also influence FP/C use negatively by accusing their female partners of salacious behaviours, as one adult female participant explained:

*“[Y] ou will speak to the person and tell him you want to use injection and he will start to have stories saying since you are on injection it means you are sleeping around. You must not tell him that you are on injection you must just go and have your injection and keep quiet.”*

*[Urban Adult Females, FGD].*

Another reported factor was the difference in discordant fertility desires between partners. One female participant reported that men generally want more children which could result in men being resistant to FP/C use:

*“I’m saying sometimes you discuss [FP use] with your partner, but other times you don’t discuss with him because sometimes males enjoy [it] that they have many children [...] Males don’t care if they had penetrated [you] and you fell pregnant. He doesn’t have stress you see.”*

*[Rural Young Adult Females, FGD]*

Desire for boy-children by men contributed to additional male resistance to FP/C use as on adult male explains:

*“[A] nother thing is that we want our families to grow. You sometimes find that you are the only boy in your family, all the others are girls. You will find that sometimes you are getting girls too, your wish is that ‘I wish to get a boy’ [ … ]”*

*[Adult Males, FGD]*

Cultural constructs about gendered roles, responsibilities and dynamics within this local setting is a contributing factor in male opposition to FP/C use. Discussions about marriage and the payment of a bride wealth (*iLobola*) highlighted this theme of gender dynamics and ownership. The role of paying *iLobola* was explained by one male participant:

*“Before, my brother, you would not have sex with a girl if you are not married or without having paid lobola you see. [ … ] As time went by things changed and they never paid attention to that. But if you go to other places [traditional Zululand] you see, where [the Zulu] culture [is] still really followed, you find that thing happening where a guy does not have sex with a girl without having paid lobola [...] If you had sex with that girl without having paid lobola it is a must my brother that he pays for her because she has become his wife.”*

*[Young Adult Males, FGD]*

Despite the changing practices surrounding marriage and the payment of bride wealth, the fact that women continue to belong to their male partners was highlighted by one key stakeholder HCP:

*“Well, definitely we need to have more male involvement because [ … ] I said women are really- they are almost like their properties, and I hate to say that [ … ]. They are the properties of the men.”*

*[Female KI, HCP, IDI]*

A female participant described how men own women’s bodies and control reproduction through the payment of bride wealth:

*“No, you not supposed to [use contraception], you have to wait for your hubby [husband], your hubby has to get it [vagina] as it is. Because they say he paid for it [vagina], isn’t it?”*

*[Females without Children, FGD]*

This ownership could result in discontinuation of FP/C use, as a married female participant explained:

*“I cannot use a condom with a woman that I am going to marry’ [imitating a male partner with a deep voice], and they have many stories.”*

*[Married Females, FGD]*

Physical abuse and concomitant contraceptive sabotage were additional ways in which men reportedly prevented female partners from using FP/C, as one adult female participant explained:

*“[T] here are people who do not do anything [use FP/C methods] and when you advise her that times are difficult she says “my partner will fight with me and my religion does not allow me to use family planning and injection. Now I am forced [not to use FP/C] because my partner is going to hit me, he is going to fight with me”.*

*[Urban Adult Females, FGD]*

Related to the physical abuse, were reported instances where male partners damaged FP/C methods, to compromise their effectiveness. A KI HCP described how some male partners break the Implant in their female partner’s arms:

*“[W] ith the Implanon [ … ] the boyfriend [s] and the husband [s], for some reasons they were finding it- feeling it- [ … ] And they were actually saying ‘go and take it out’, they were actually breaking it in the woman’s arm [ … ] But with the Implanon I guess they knew that it was set underneath the skin so then they were going and actually feeling for it and they found it and then it became an issue.”*

*[KI, HCP, IDI]*

A female participant explained how men sometimes punctured male condoms to establish power in relationships:

*“Look you hold a male condom [ … ] at the beginning and put it on, he pretends as if he is holding it, yet he [tears] it you see [ … ] It has a hole and since it has a hole this boy wants a baby...”*

*[Rural Adult Females, FGD]*

A major theme that emerged from this data was covert use of FP/C methods by female partners because of male partner opposition. Covert use was widely discussed by all the participants in the study, suggesting that this behaviour is perhaps more prevalent in this setting than elsewhere. It was often linked to a lack of communication between men and women.

*“At first you will think that you are respecting him by discussing with him, then it’s just that boys don’t like the issue of injections. Then you see that it’s better to just go the clinic without telling him, but most of the boys really don’t like this thing that’s why we are hiding it from them when we [are] doing it.”*

*[Rural Adolescent Females, FGD]*

A HCP added to the covert use conversation by describing that women hide their clinic cards from their male partners:

*“I think it is a problem that a man does not want his partner to do family planning, they say ‘sister I hide my card because he does not want me to do family planning because he thinks I am having many partners’.”*

*[HCP Group 2, FGD]*

A behaviour that was confirmed by a female participant who reported using FP/C methods:

*“I go for injection come back and hide my card, on my date I go and come back there at the clinic, I don’t tell him.”*

*[Females without Children, FGD]*

One adult female participant explained how male misinformation, side effects and covert use link together in this setting:

*“I am going to agree with them because it is right if you start by going for injection without telling him because he never feels anything but once you tell him he is going to talk about water that he never felt before [yes, laughing]. Maybe you have been having sex for a year while on injection. Now that you have told him about injection. If you want to see how much a man loves you tell him about injection issue [laughing] he will tell you that you are cold, or you have water [somebody laughing] but you have been having sex without him feeling it”*

*[Urban Adult Females, FGD]*

### The supportive male partner

As the quotes from the female participants below point out, not all male partners are the same. Some male partners were described as sources of information and being supportive and encouraging of FP/C use.

*P1: “No there is no such a thing [in response to previous comment that men obstruct FP/C use], mine doesn’t have a problem, he even reminds me that ‘no your date [is] like this and that, do you still remember’, he doesn’t have a problem about water [being wet] [ … ], no he is right [ … ] For me it was him who took me to the clinic”.*

*P4: “[ … ], mine he takes me to the clinic, he accompanies me to go for injection if he is around. He likes that I take injection, he can’t see himself without me taking injection.”*

*[Rural Young Adult Females, FGD]*

A quote from one young male adult further supports this view:

*“Plan the family by first going to the clinic to get counselling with your partner. You go with your partner to counselling session [s] [ … ] and they will explain further about that thing.”*

*[Young Adult Males, FGD]*

Numerous female participants reported that their male partners were often their source of FP/C information:

*“I have a great relationship with my boyfriend. He is the one who told me that ‘no baby, have you heard about this 3 years [implant] that is now available?’ I said ‘what 3 years now?’, he said ‘we have to get protected even though we are using the first one [male condoms] let’s use this one as well’ [ … ]”*

*[Urban Young Adult Females, FGD]*

Some female participants remarked that there were few sources of support in their communities for FP/C use where they lived, and that their male partners often provided this support:

*“Most of the time you get it [support] from males, your partner than women. Women, other women, when you say that you [are] on contraceptives others criticise you [...] And [you] find that they criticise you. Friends criticise you, not saying it to you, talking to others saying you [are] on injection, so and so. You see, they criticise you. [F: So, there is no support in the community?] It is not there in the community.”*

*[Females without Children, FGD]*

Male partners also play a role in reminding women to take their FP/C methods, as one female from the married FGD group described:

*“I used them [Pill] for 6 years and was reminding myself with Generations [local television series]. The nice thing is my partner used to call and remind me, [ … ] Because he knew that I was using pills, when I visit him he will ask if I brought them and if I did not we will go back and fetch them [all laughing ] he used to remind me and it was nice because we used to remind each other.*

*[Married Females, FGD]*

Another female participant added to this:

*“I was told by my boyfriend and even now he reminds me, he asks me that, ‘have you gone for injection?’, because we now have a lot of kids”*

*[Urban Young Adult Females, FGD]*

This support resulted in improved access to FP/C services for women, as one young adult female explained:

*“My partner asked me if I did go for family planning on this day, I said ‘oh I did not go you know, I forgot’. We went with him the following day.”*

*[Rural Young Adult Females, FGD]*

One female participant explained that such behaviour showed that these male partners shared the responsibility of family planning.

*P: “For me it was him who took me to the clinic [all laughing]”.*

*I: “What did he say you are going to do?”*

*P: “He said I must go take injection to prevent getting another child, we are still young. I said okay, he is wise he got brains.”*

*[Rural Young Adult Females, FGD]*

Sharing the responsibility of using FP/C was a key facilitating factor in FP/C use, as described in the quotes above and below. A male participant shared this view of the importance of shared responsibility:

*“There is something that recently happened, and I liked it, it made me happy. There is a guy I am friends with, [ … ] he asked one female we were hanging out with to help accompany him to go buy the morning after pills. You see us as males if, we cannot just leave things to them [females] saying that they must do certain things, you too if you know that there is a certain mistake that you made, that courage you give yourself that you see at least you ask because that means the female was going to go, but he is the one who stood up [and said] that because we did this let me go,[ … ]. All of us should not just leave things [FP/C use] to women as if they are the ones one-way [get pregnant alone], it is important for us males to think as well.”*

*[Adult Males, FGD]*

## Discussion

The uptake and utilization of FP/C methods and services is key to improving unmet need and decreasing unintended or unplanned pregnancies. In this paper the role of men in FP/C uptake and use was explored within the urban/peri-urban and rural/peri-urban setting in the eThekwini district of KwaZulu-Natal from a community and HCP perspective.

Use of chronic or long-term medical interventions such as FP/C methods fluctuate on a continuum of use, resulting in variation of use patterns [43]. The findings from this study showed that the influence of the male partner can result in varied degrees of FP/C use. Three predominant outcomes on use were reported namely: discontinuation, covert, and improved FP/C uptake and use. Contextualisation of findings and awareness of the various macro-structural forces behind male influence on FP/C use is important for increasing the uptake and continued use of FP/C.

The use of FP/C methods within marriage continues to be a focal point of policy and research within the sub-Saharan setting. However, unlike other countries in the Sub-Saharan region, South Africa is experiencing a decline in marriage rates with KwaZulu-Natal, where this data was collected, having some of the lowest marriage rates in the Sub-Saharan region [37]. In this study, no male participants reported being married (or had ever been married), and only 3% of female participants reported being married. The decline in marriage rates has implications for FP/C use monitoring, interventions and programs. The CPR for unmarried women in South Africa decreased from 68% in 1998 to 64% in 2016, and unintended pregnancies are strongly associated with single or divorced people [3, 10].

Furthermore, the changes in this fundamental cultural practice has implications for the gender dynamics in relationships. Male participants in this study mentioned how guiding cultural practices involving reproduction are changing. These changes have resulted in men and women renegotiating reproductive behaviour, especially with reported behaviours such as covert FP/C use and male partner abdication of childcare responsibilities. Further research is needed to explore the importance and influence that the change in this fundamental cultural practice may have on the uptake and use of FP/C.

Another key finding from this data was the high rates of unplanned pregnancies reported. Most of the community female and male participants reported that their pregnancies were unplanned, despite having knowledge and access to FP/C services and methods. This finding is in keeping with findings from other studies that investigated unplanned pregnancies [9, 13]. The findings from this study sheds some insight into reasons for non-FP/C use that could result in unplanned pregnancies. In particular the role of the male partner as a contributing factor to non-use of FP/C was explored.

### Discontinuation of FP/C use

Most of the negative factors that result in barriers to use overlap in their influence and extent in which they result in FP/C discontinuation. Some of the opposing factors resulted in covert, or interrupted use, whereas others led to more permanent discontinuation of FP/C use. Factors linked to discontinuation included limited understanding about FP/C methods, side-effects (real or perceived), gender power dynamics, physical abuse, and FP/C sabotage.

Gender power dynamics continue to play a crucial role in the use of FP/C methods and services, as demonstrated by this study and others [8, 16, 30, 48]. Power imbalance was reported more in the marriage setting, where men assumed ownership over female fertility, but the responsibility of raising children belonged to women. In addition to this, discordant fertility desires were also reported with men preferring more children than women. In this context of commodified fertility and reproduction, female partners were subjected to male partner demands and expectations for children. These findings were not unique to this study and was previously reported [8, 28, 49].

Accounts where female participants and HCPs described physical abuse and fear of the male partner were described as reasons to permanently discontinue FP/C use. Female participants described how women may not initiate FP/C use or discontinue use out of fear that their male partners will physically abuse them. This fear is not unfounded since physical abuse is prevalent in this setting where 21% of ever partnered women had experienced abuse by their partners [3].

FP/C sabotage was considered another form of abuse reported by all the participants that resulted in discontinued use. HCPs reported instances where male partners would feel for the hormonal Implant and break it in their female partner’s arm. Community participants described how men and boys intentionally break or damage male condoms to prevent FP/C use. These reports link male opposition to discontinued method use through physical abuse and is a serious concern for FP/C uptake and use, which has been noted elsewhere [50].

### Covert and interrupted FP/C use

An important finding from this data was that negative male partner influence did not always result in total discontinuation of FP/C use, but also resulted in modified behaviour by female partners, who continued using FP/C without their male partner’s knowledge. Findings from this study revealed that in this setting, covert use may be a common practice amongst female partners and was widely encouraged by female participants and healthcare providers. The extent of covert FP/C use remains largely unknown as this data is not routinely collected [51, 52]. Other studies from the sub-Saharan region found that covert use was not widely practiced [2, 27].

Female participants and HCPs reported that covert use was a means for women to control their own FP/C choices without the permission or knowledge of their male partners. Despite the seemingly positive influence of this, covert use should be treated with caution as it can result in inconsistent or discontinued use [53]. The fact that women use FP/C covertly in this setting suggests that they have limited negotiating power within their relationships, highlighting their vulnerability.

Providing adequate, accurate and contextually acceptable information is crucial to improving men’s attitudes and understanding towards FP/C use. Despite the 2016 SADHS reporting that most people of reproductive age in SA has some FP/C information, the data from this study showed that the current provision of FP/C information is inadequate [3]. Most male participants lacked a clear understanding about FP/C methods, their mechanism of action and related side-effects. This resulted in misconceptions and myths about side-effects and the reported concern that FP/C methods could harm male partners. HCPs explained that the lack of male understanding can be attributed to the feminisation of FP/C programmes, where the focus remains on women. According to female participants these misunderstandings resulted in misconceptions and myths related to FP/C use, such as female partner infidelity and excessive vaginal wetness.

Side-effects remain a widely reported barrier to FP/C use, in this study and others [26]. Male participants described side-effects as a key reason why they do not like or encourage FP/C use. Common side-effects reported by male partners include decreased male sexual pleasure, reduced libido, abnormal menstrual bleeding, and increased vaginal lubrication or wetness. Side-effects can be linked to inadequate information, cultural constructs about reproductive behaviour, male sexual entitlement, and gender power inequality [6, 20, 54]. Resistance to FP/C use by male partners are also reported in other studies [27, 29, 54, 55].

Male and female participants reported that increased vaginal wetness, whether real or perceived, was a reason for male partners to discourage FP/C use. However, male and female participants had differing views about whether increased vaginal wetness due to FP/C use was an actual problem. Female participants felt that men constructed and exacerbated reports of vaginal wetness to encourage discontinuation of FP/C use. Male participants reported it as a real concern for them as it decreased their sexual pleasure. Increased vaginal wetness has been reported previously within the South African context before and it is linked to discontinued FP/C use [54, 56, 57].

Decreased male sexual pleasure was another reported barrier to FP/C use. Male condom use reportedly decreased male sexual pleasure the most, followed by increased vaginal wetness from hormonal FP/C methods (especially the injection). The 2016 SADHS results show that 58% of women and 65% of men use male condoms inadequately during high risk sexual practices [3]. The importance of decreased male sexual pleasure on FP/C use, especially male condoms, requires further exploration, and is reported in other studies in this setting [55, 58].

### Improved access, uptake and adherence to FP/C use

Much has been written about the opposing influence that men can have on FP/C use. Less is known about the supportive role and influence that men can have on FP/use. This study highlighted that male support for FP/C can help female users to overcome barriers to using FP/C by facilitating access, encouraging uptake, and improving adherence to FP/C methods.

### Improved access

In this study, male and female participants reported that males accompanied their female partners to their local clinics to obtain their FP/C methods. This differs from other studies where male accompaniments to FP/C facilities are ridiculed and FP/C is considered only a female domain [28]. Men are generally economically more empowered which enables them to overcome access barriers such as transport costs or healthcare related fees [7]. By accompanying their female partners, they provide financial support to improve access to FP/C services [28, 53].

Male partners also provided social support for women to access FP/C services. Community related stigma can negatively impact on FP/C use, especially if unmarried or adolescent women are accessing FP/C services within their immediate community. Female participants reported that little support for FP/C use exists in local communities and this negatively impacts on them seeking FP/C services and methods. In this sense, the male partner accompanying women to clinics can mitigate against community related stigma.

### Encouraging uptake of FP/C

Female participants reported that in certain cases their male partners suggested and initiated the use of FP/C methods. This point highlights the need for men to be included in FP/C services and programmes [53, 59]. Men have limited FP/C methods available to them and rely on their female partners to use FP/C methods to prevent unplanned pregnancies. Although this may be viewed as a limiting factor, it may result in men encouraging female partners to use FP/C methods to meet their own needs [59].

The encouragement of FP/C use by men was positively viewed by male and female participants and reflects the recognition by men that cultural and patriarchal practices and attitudes are changing. Cultural change can happen slowly and over many years [23], but if those changes have a positive outcome, then the effects can be tremendous. More culturally focused FP/C programming can greatly assist in constructing new ways in which men and women engage about FP/C use.

Male participants pointed out how their perceptions and behaviour regarding FP/C use is changing. This may be linked to changes in marriage practices in this setting. According to these men, traditional cultural practices are still strictly observed in more rural areas, whereas is less constrained in peri-urban and urban settings. Contemporary men and women in this setting are negotiating and constructing new roles that they occupy within this cultural context, whereby women take ownership of their reproductive health, and men adapt their behaviour to encourage FP/C use.

### Improved adherence

Improved adherence to FP/C is probably one of the key positive influencing factors that men can have on FP/C use which was reported by female participants. Forgetfulness is a key barrier to continued adherence to any long term chronic treatment. Social support is especially important to ensure that adherence remains adequate and is sustained, especially when negative side-effects can threaten continued use. As demonstrated by this data, men can and do play a key role in improving adherence by reminding their female partners to take FP/C methods, and to attend their scheduled visits at the clinic.

### Limitations

While we deem the methodology, sampling size, and strategies used in this study as adequate, some limitations should be noted. Commonly in qualitative studies limited sample size and generalizability of the findings are critiqued [60]. The aim of qualitative research is in depth and description, and not necessarily breadth [61, 62]. Therefore, we should apply caution in generalizing the findings. The data from this study has shown how important contextualisation is when exploring FP/C use. Sometimes not being able to return to the field to follow up on findings, places a limit to the depth of exploration of themes, but returning into the field has cost and time implications. One way to compensate for this limitation is to sample from a variety of participants within a community [63]. Variation sampling was achieved in this study where a variety of participants ranging from community members to healthcare providers were interviewed to obtain a robust exploration to the study question.

## Conclusion and recommendations

In this paper we have shown how men influence the uptake, use and discontinuation of FP/C methods and services in the contemporary South African setting. The focus was on how men influence FP/C use as either a barrier or facilitator. These two opposing views provide insight into the complex role that the male partner plays in SRH in general in the South African setting. The preconceived and often perpetuated notion that men are always barriers to FP/C use in this setting is challenged by the findings presented in this paper.

Socially constructed gender dynamics continue to play a key role in FP/C uptake and use. Changes in cultural practices linked to gender dynamics has resulted in men and women renegotiating the FP/C use space. This was seen in the discussions about covert FP/C use, the decline in marriage, and the influence of side effects on sexual pleasure. These findings, and especially the decline in marriage, needs further exploration in the context of FP/C uptake and use.

The other key factor that was highlighted and needs to be further explored is providing accurate, contextual and culturally acceptable information to men to engage them positively into FP/C care. While men had good levels of information about FP/C products, they lacked a clear understanding about how these products work and their related side effects. There is a need to reconsider the manner in which FP/C information is delivered to men to improve their understanding and active participation in FP/C uptake and use.

The findings from this paper have implications for national policy and public health FP/C programmes. In particular, it was highlighted that although the current family planning policy acknowledges that men should be more included in reproductive health, they are largely excluded in reality. Little guidance is provided in the current FP/C policy as to how male involvement can be improved. The findings from this study can be useful in exploring and developing strategies that can improve male partner involvement in FP/C use.

Of importance is the need to provide sufficient information and counselling to men and to build on the positive strategies through which men can support and promote FP/C use as described in this paper. It is crucial for FP/C services to be more inclusive of the male partner interaction. A key strategy as highlighted by the data in this paper is to promote discussions between female and male partners about FP/C use and pregnancy intentions that will be empowering for both the partners.

While numerous studies have outlined high rates of unplanned pregnancies, few explore the reasons behind non-use of FP/C that result in unplanned pregnancy. The findings from this study focused on one particular factor, namely the male partner.

Finally, this paper has shown the importance and usefulness of a community approach when investigating sexual and reproductive health. The involvement of men has added a unique and much needed perspective on FP/C uptake and use in the South African setting. Using a community-based approach to inform the development of FP/C research and interventions could be a possible solution to develop improved FP/C programmes and services. The community-based approach followed in this study highlighted the potential effectives of engaging men.

## Data Availability

The datasets generated and/or analysed during the current study are not publicly available due the sensitive nature of the data and being qualitative there is a high risk of compromising participant and healthcare system confidentiality but are available from the corresponding author on reasonable request.

## Abbreviations

CPR: Contraceptive prevalence rate
CYPR: Couple Year Protection Rate
FGD: Focus Group Discussion
FP/C: Family planning and contraception
HCP: Healthcare Provider
HIV: Human Immunodeficiency Virus
IDI: In-depth Individual Interviews
KI: Key Informant/stakeholder
SA: South Africa
